# Development and Hybrid Position/Force Control of a Dual-Drive Macro-Fiber-Composite Microgripper

**DOI:** 10.3390/s18041301

**Published:** 2018-04-23

**Authors:** Jin Zhang, Yiling Yang, Junqiang Lou, Yanding Wei, Lei Fu

**Affiliations:** 1 Faculty of Mechanical Engineering and Mechanics, Ningbo University, Ningbo 315211, China; zhangjin08@nbu.edu.cn; 2 The Key Laboratory of Advanced Manufacturing Technology of Zhejiang Province, Zhejiang University, Hangzhou 310058, China; weiyanding@yahoo.com (Y.W.); fu-lei@zju.edu.cn (L.F.); 3 State Key Laboratory of Fluid Power and Mechatronic Systems, Zhejiang University, Hangzhou 310058, China

**Keywords:** micro-fiber-composite, microgripper, hybrid control, micromanipulation, position/force

## Abstract

This paper reports on the development, implementation and hybrid control of a new micro-fiber-composite microgripper with synchronous position and force control capabilities. In particular, the micro-fiber-composite actuator was composed of rectangular piezoelectric fibers covered by interdigitated electrodes and embedded in structural epoxy. Thus, the micro-fiber-composite microgripper had a larger displacement-volume ratio (i.e., the ratio of the output displacement to the volume of the microgripper) than that of a traditional piezoelectric one. Moreover, to regulate both the gripper position and the gripping force simultaneously, a hybrid position/force control scheme using fuzzy sliding mode control and the proportional-integral controller was developed. In particular, the fuzzy sliding mode control was used to achieve the precision position control under the influence of the system disturbances and uncertainties, and the proportional-integral controller was used to guarantee the force control accuracy of the microgripper. A series of experimental investigations was performed to verify the feasibility of the developed microgripper and the control scheme. The experimental results validated the effectiveness of the designed microgripper and hybrid control scheme. The developed microgripper was capable of precision and multiscale micromanipulation tasks.

## 1. Introduction

**Figure 1:**
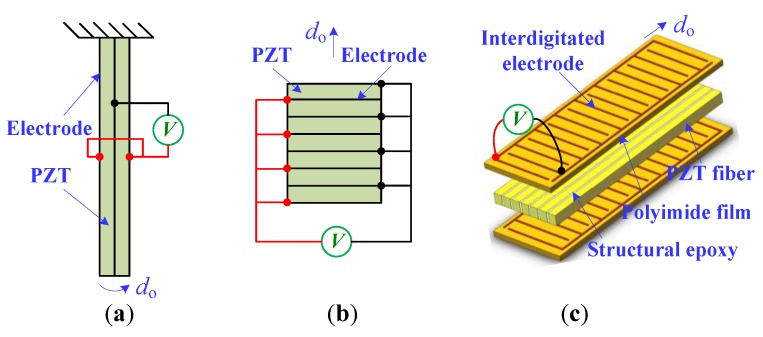
Schematic diagram of piezoelectric actuators: (**a**) the PBA; (**b**) the PSA, and (**c**) the MFC actuator.

During the past few decades, the piezoelectric actuator has received much attention in the fields of micromanipulation and microassembly [1,2,3]. This is because piezoelectric material exhibits high resolution and fast response capabilities [4,5]. In particular, piezoelectric actuators are involved in various types of bending actuators (*d*_31_ effect, i.e., the material deformation direction is perpendicular to the polarization direction), stack actuators (*d*_33_ effect, i.e., the material deformation direction and polarization direction are the same), and macro-fiber-composite (MFC) actuators (*d*_33_ effect), as illustrated in Figure 1. When a driving voltage is applied to the piezoelectric bending actuator (PBA), the piezoelectric ceramic (PZT) will elongate or shorten in the length direction [6]. Thus, the material body (e.g., the beryllium bronze and carbon fiber) will cause the bending deformation of the mechanism. Moreover, the piezoelectric stack actuator (PSA) is composed of a multilayer PZT substrate. Its structure is mechanically connected in series and electrically in parallel, as shown in Figure 1b. In addition, the MFC actuator consists of rectangular PZT fibers sandwiched between electrodes, polyimide film, and layers of adhesive. Therefore, the MFC actuator prevails, owing to its flexible nature, large outputs, and damage tolerance [7].

To effectively manipulate micro-objects ranging in size from micrometers to millimeters, both high resolution and a large workspace range are desirable for microgrippers in a microhandling system [8,9]. To date, many microgrippers driven by the PBA and PSA have been reported. However, the PBA usually has a small output displacement (approximately 20 μm). Even when combined with another actuation source (e.g., the thermal actuation), the stroke of the PBA-driven microgripper is no more than 100 μm [10]. Although the PSA-driven microgripper can produce a large gripping range, complex compliant mechanisms and huge structural dimensions are needed. For example, the authors in [11] reported a PSA-driven microgripper based on the double-rocker mechanism where the output displacement was 427.8 μm. However, the microgripper also had huge geometric parameters, resulting in an inferior displacement-volume performance. Considering that the output displacement has to be compared with the global size of the microgripper [12], this research focused on the development of a microgripper driven by a MFC actuator. When compared with the PBA and PSA, the MFC actuator can provide a large output displacement, which simplifies the gripper structure and decreases the number of amplification flexures.

However, in most studies on MFC actuators, the operation accuracy is lower than the millimeter level. The extension of the MFC actuator to anultrahigh position control is rarely considered, except for in [13], where a feed forward control based on the Bouc-Wen model and a linear feedback control were used to compensate the hysteresis nonlinearities arising from the inherent characteristics of the MFC actuator. However, a single MFC manipulator was investigated, and only the position control was conducted. Generally, the synchronous control of the gripper position and the gripping force is extremely challenging, but is essential in precision micromanipulation tasks [14]. In [15,16], a switching control scheme was proposed to regulate the position and force of the piezoelectric microgripper in an alternate manner. However, the two control variables were switched successively. It was difficult to obtain a smooth transition, and only the position or the force could be well controlled in each phase. The disadvantages of the switching control could be avoided using the impedance control scheme. Based on the Lyapunov approach, [17] developed a position-based impedance control and a sliding mode impedance force control for a PBA-driven microgripper. In [18], a discrete impedance control for both the gripper position and gripping force was proposed, and the control stability was verified by introducing an appropriate Lyapunov function.

Nevertheless, the impedance control scheme only uses one actuator to regulate two variables. It is a typical under-actuated system. Thus, the control accuracy presents a compromise between the position and force [19]. To achieve a balance between the two variables, the control accuracy of the impedance control scheme is necessarily degraded. On the other hand, hybrid control using two independent actuators is suitable for the simultaneous control of the position and force [10]. As for the control law, the sliding mode control (SMC) presents great potential in stable and precise control applications [20,21,22]. However, the switching gain in most SMCs is constant or quasi-linear. Concerning system disturbances and model uncertainties in the microgripper, a SMC with a nonlinear adaptive law is desirable [23,24].

To this end, a new MFC microgripper with a large displacement-volume ratio was developed. Meanwhile, a hybrid control scheme was designed to realize the simultaneous control of both the position and gripping force. In particular, a new MFC microgripper with two independent MFC actuators is presented. Moreover, the position of the right gripping arm was determined using a fuzzy sliding mode control (FSMC), and the force of the left gripping arm was regulated by a PI controller. Several experimental investigations were conducted to demonstrate the efficiency and the feasibility of the developed microgripper and the control scheme.

The rest of this paper is organized as follows. The description of the MFC microgripper is presented in Section 2. Section 3 shows the hybrid control scheme for regulating the position and the gripping of the microgripper. The prototype development, a series of experimental studies, and the discussions are outlined in Section 4. In Section 5, the conclusions are described.

## 2. Description of the MFC Microgripper

**Figure 2:**
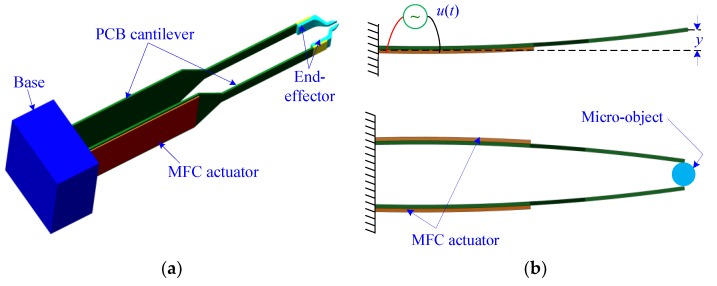
(**a**) Schematic diagram of the MFC microgripper; (**b**) Illustration of a micro-object gripping with a microgripper that contains a pair of MFC actuators.

The CAD drawing of the MFC microgripper is shown in Figure 2. As illustrated in Figure 2, the developed microgripper consisted of a pair of MFC actuators, a pair of printed circuit board (PCB) cantilevers, a pair of end-effectors, and a support base. Moreover, two identical gripping arms were used to construct the microgripper, and each gripping arm was composed of one MFC actuator (model: M2814-P1, from Smart Material Corp, Sarasota, FL, USA), one PCB cantilever, and one end-effector. Furthermore, the MFC actuator was attached to the base end of the PCB cantilever using epoxy (model: DP460, from 3M Company, Maplewood, MN, USA). The gripping arm could generate a large output displacement at the tip end when an input voltage was applied to the MFC actuator. As the microgripper was driven using two separate actuators, the gripping arm could be controlled independently, which provided a more dexterous and reliable micromanipulation operation. In addition, by employing one gripper arm with a MFC actuator as a basic module, a multi-modular gripper could be easily developed.

The end-effector was glued onto the front end of the PCB cantilever, as shown in Figure 2a. Thus, the structure and material of the gripper tip could be adapted to the shape of the micro-object by merely changing the end-effectors. Hence, the microgripper system became simple, and the effectiveness of the manipulation applications could be improved. The end-effectors were manufactured using a stereo lithography apparatus (model: RS6000, from Uniontech three-D Technology Co., Ltd., Shanghai, China) along with various materials of a methacrylate photosensitive resin (from Formlabs Corporation, Somerville, MA, USA). Furthermore, the MFC actuator had a very flexible nature and a larger input voltage range from −500 V to 1500 V, while the maximal voltage applied to the conventional piezoelectric actuator was lower than 200 V. In addition, the MFC actuator had a greater electro-mechanical coupling coefficient than the conventional one. Therefore, the MFC actuator could provide a larger output displacement and a higher actuation force, which makes the MFC actuator suitable for use in the actuation of a microgripper dedicated to multiscale micromanipulation applications.

## 3. Hybrid Position/Force Controller Design

### 3.1. Position Controller Design for the Right Gripping Arm of the Microgripper

The purpose of the position controller is to regulate the driving voltages applied to the MFC actuator once the desired position trajectories of the gripping arm are specified. A FSMC based on the dynamic model combined with a fuzzy regulator was used to determine the output displacement of the microgripper. Therefore, the position control performance was guaranteed by the FSMC.

It is known that the dynamic model of the piezoelectric system can be presented as follows:

(1) $$\{\begin{array}{l} m\ddot{y}(t)+b\dot{y}(t)+cy(t)=c\left[d_{p}u(t)-ℋ(t)\right]+p(t)\\ \dot{ℋ}=\alpha d_{p}\dot{u}-\beta \left|u\right|ℋ{\left|ℋ\right|}^{n-1}-\gamma u{\left|ℋ\right|}^{n} \end{array}$$

where the parameters *m*, *b,* and *c* denote the equivalent mass, the damping coefficient, and the stiffness of the microgripper, respectively; *y* is the tip displacement as shown in Figure 1b; *u* represents the control voltage; *t* is the time variable; *p*(*t*) denotes the perturbation term arising from creep, external force, system disturbances, and model uncertainties; ℋ denotes the nonlinear displacement hysteresis, and its shape and magnitude is determined by parameters *α*, *β*, *γ*, and *n*; and *d_p_*represents the piezoelectric coefficient and the driving voltage of the MFC actuator. Considering that the structure of the microgripper is flexible, the parameter *n* was assigned as one [13].

Supposing that the position error *e_y_*(*t*) = *y_d_*(*t*) −*y*(*t*), where *y_d_*(*t*) is the desired signal and *y*(*t*) denotes the actual signal. 

Then, the sliding function can be defined as

(2) $$s\left(t\right)=\lambda_{P}e_{y}\left(t\right)+\lambda_{I}\int_{0}^{t}e_{y}\left(\tau \right)d\tau +{\dot{e}}_{y}\left(t\right)$$

where *λ_P_* and *λ_I_* are designated positive parameters.

Note that the equivalent control *u_eq_* is the solution to ${\dot{s}}_{r}$(*t*) = 0, the equivalent control can be derived by combining Equations (1) and (2).

(3) $$\begin{array}{ll} u_{eq} & =\frac{m}{cd_{p}}[\left(\frac{c}{m}-\lambda_{I}\right)y+\left(\frac{b}{m}-\lambda_{P}\right)\dot{y}+{\ddot{y}}_{d}\\ & +\lambda_{P}{\dot{y}}_{d}+\lambda_{I}y_{d}+\frac{c}{m}ℋ-\frac{p_{\mathrm{est}}}{m}] \end{array}$$

where *p*_est_(*t*) represents the estimation value of the perturbation term *p*(*t*), which can be acquired by its one-step delayed estimation [17].

(4) $$\begin{array}{ll} p_{\mathrm{est}}(t) & =m\ddot{y}(t-T)+b\dot{y}(t-T)+cy(t-T)\\ & -cd_{p}u(t-T)+cℋ(t-T) \end{array}$$

Considering that sometimes the initial force of the microgripper does not lie on the sliding surface, and system disturbances and uncertainties always exist, an extra reaching law *u*_sw_ is needed. Thus, the total control can be expressed as

(5) $$\begin{array}{ll} u_{y} & =u_{eq}+u_{sw}\\ & =\frac{m}{cd_{p}}[\left(\frac{c}{m}-\lambda_{I}\right)y+\left(\frac{b}{m}-\lambda_{P}\right)\dot{y}+{\ddot{y}}_{d}+\lambda_{P}{\dot{y}}_{d}\\ & +\lambda_{I}y_{d}+\frac{c}{m}ℋ-\frac{p_{est}}{m}+\eta \mathrm{sgn}\left(s_{r}\right)+\delta s_{r}] \end{array}$$

where *δ* (*δ*>0) is a control gain; *η* (*η*> 0) denotes a positive switching gain; *T* is the sampling time; and sgn(∙) represents the sign function.

**Theorem** **1.** *For the system (1) with the sliding function (2), the sliding mode will occur in afinite time if control law (5) is used.*

**Proof.**  The Lyapunov function candidate is chosen as

(6) $$ℒ=\frac{1}{2}s_{r}{}^{2}$$

Then, the following conditions are required:

(7) $$\dot{ℒ}=s_{r}{\dot{s}}_{r}<0$$

The first derivate of the sliding function *s* can be written as

(8) $$\begin{array}{ll} {\dot{s}}_{r} & =\left(\frac{c}{m}-\lambda_{I}\right)y+\left(\frac{b}{m}-\lambda_{P}\right)\dot{y}+{\ddot{y}}_{d}+\lambda_{P}{\dot{y}}_{d}\\ & +\lambda_{I}y_{d}+\frac{c}{m}ℋ-\frac{P_{\mathrm{est}}}{m}-\frac{P_{\mathrm{err}}}{m}-\frac{cd_{p}}{m}u_{y} \end{array}$$

where *p*_err_(*t*) is the perturbation estimation error, i.e., *p*_err_(*t*)= *p*(*t*) −*p*_est_(*t*).

Combining Equations (2), (6), and (8), we have

(9) $$\begin{array}{ll} \dot{ℒ} & =s_{r}{\dot{s}}_{r}\\ & =s_{r}[\left(\frac{c}{m}-\lambda_{I}\right)y+\left(\frac{b}{m}-\lambda_{P}\right)\dot{y}+{\ddot{y}}_{d}+\lambda_{P}{\dot{y}}_{d}\\ & +\lambda_{I}y_{d}+\frac{c}{m}ℋ-\frac{p_{\mathrm{est}}}{m}-\frac{p_{\mathrm{err}}}{m}-\frac{cd_{p}}{m}u_{y}]\\ & =s_{r}\left[-\eta \mathrm{sgn}\left(s_{r}\right)-\delta s_{r}-\frac{p_{\mathrm{err}}}{m}\right]\\ & =-\eta \left|s_{r}\right|-\delta s_{r}{}^{2}-\frac{p_{\mathrm{err}}}{m}s_{r} \end{array}$$

When the switching gain *η* is designed to meet the condition as follows

(10) $$\eta >\frac{\left|P_{\mathrm{err}}\right|}{m}+\kappa$$

where *κ* (*κ*> 0) denotes an arbitrary constant.

Then, the following equation is derived

(11) $$\begin{array}{ll} \dot{ℒ} & =s_{r}{\dot{s}}_{r}\\ & =-\eta \left|s_{r}\right|-\delta s_{r}{}^{2}-\frac{p_{\mathrm{err}}}{m}s_{r}\\ & <-\eta \left|s_{r}\right|-\frac{p_{\mathrm{err}}}{m}s_{r}\\ & <-\kappa \left|s_{r}\right|<0 \end{array}$$

Therefore, Equation (7) can be satisfied using Equations (9)–(11). According to the Lyapunov stability theorem, the control system is stable, and the states reach the sliding surface in a finite time.

When using the SMC to conduct the position tracking, the chattering phenomenon may occur, since the function sgn(*s_r_*) in Equation (5) is discontinuous. Thus, a saturation function was used to substitute the sign function to suppress the chattering. The saturation function is given by

(12) sat(sr)={1sr>εsrε|sr|<ε−1sr<−ε

where *ε* (*ε*> 0) denotes the thickness of the boundary layer. It was observed that a linear feedback control was used when *s_r_*was within the boundary layer, and the switching control was only employed if *s_r_*was outside the boundary layer.

Moreover, a fuzzy regulator was used to adjust the switching gain *η* in terms of the system uncertainties. According to the SMC control process, the switching gain *η* should increase when the position trajectory has not reached the ideal sliding surface (i.e., $s_{r}{\dot{s}}_{r}$> 0). Furthermore, the gain *η* needs to decrease if the trajectory has crossed the sliding surface (i.e., $\;s_{r}{\dot{s}}_{r}$< 0). Consequently, a fuzzy regulator can be developed. The fuzzy sets are given by

(13) $$s\dot{s}=\Delta \eta =\left\{\begin{array}{cccc} \mathrm{NB} & \begin{array}{cc} \begin{array}{cc} \mathrm{NM} & \mathrm{NS} \end{array} & \mathrm{ZO} \end{array} & \begin{array}{cc} \mathrm{PS} & \mathrm{PM} \end{array} & \mathrm{PB} \end{array}\right\}$$

where parameter $s\dot{s}$ is the linguistic variable ($s\dot{s}$= *k*_1_$s_{r}{\dot{s}}_{r}$, and *k*_1_ is the normalizing factor), the incremental Δ*η* denotes the output linguistic variables; NB, NM, and NS represent negative big, negative medium and negative small, respectively; ZO is zero; PS, PM, and PB are positive small, positive medium and positive big, respectively. 

**Figure 3:**
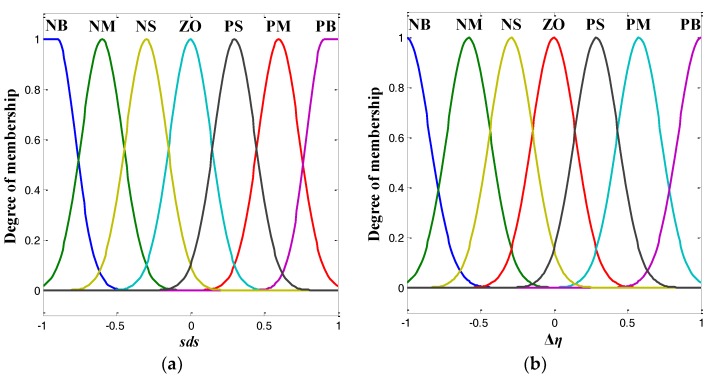
Membership functions for (**a**) the input linguistic variable, and (**b**) the output linguistic variable.

The membership functions for the input and the output linguistic variables are illustrated in Figure 3. As shown in Figure 3, Gaussian-shaped membership functions were used for the purpose of smooth transition.

In addition, the complete rule base of the fuzzy regulator can be designed as follows

(14) $$\left\{ \begin{array}{l} \begin{array}{cc} R1: & \begin{array}{cccc} \mathrm{IF} & s\dot{s} & \mathrm{is} & \begin{array}{cccc} \mathrm{PB} & \mathrm{THEN} & \Delta \eta & \begin{array}{cc} \mathrm{is} & \mathrm{PB} \end{array} \end{array} \end{array} \end{array}\\ \begin{array}{cc} R2: & \begin{array}{cccc} \mathrm{IF} & s\dot{s} & \mathrm{is} & \begin{array}{cccc} \mathrm{PM} & \mathrm{THEN} & \Delta \eta & \begin{array}{cc} \mathrm{is} & \mathrm{PM} \end{array} \end{array} \end{array} \end{array}\\ \begin{array}{cc} R3: & \begin{array}{cccc} \mathrm{IF} & s\dot{s} & \mathrm{is} & \begin{array}{cccc} \mathrm{PS} & \mathrm{THEN} & \Delta \eta & \begin{array}{cc} \mathrm{is} & \mathrm{PS} \end{array} \end{array} \end{array} \end{array}\\ \begin{array}{cc} R4: & \begin{array}{cccc} \mathrm{IF} & s\dot{s} & \mathrm{is} & \begin{array}{cccc} \mathrm{ZO} & \mathrm{THEN} & \Delta \eta & \begin{array}{cc} \mathrm{is} & \mathrm{ZO} \end{array} \end{array} \end{array} \end{array}\\ \begin{array}{cc} R5: & \begin{array}{cccc} \mathrm{IF} & s\dot{s} & \mathrm{is} & \begin{array}{cccc} \mathrm{NS} & \mathrm{THEN} & \Delta \eta & \begin{array}{cc} \mathrm{is} & \mathrm{NS} \end{array} \end{array} \end{array} \end{array}\\ \begin{array}{cc} R6: & \begin{array}{cccc} \mathrm{IF} & s\dot{s} & \mathrm{is} & \begin{array}{cccc} \mathrm{NM} & \mathrm{THEN} & \Delta \eta & \begin{array}{cc} \mathrm{is} & \mathrm{NM} \end{array} \end{array} \end{array} \end{array}\\ \begin{array}{cc} R7: & \begin{array}{cccc} \mathrm{IF} & s\dot{s} & \mathrm{is} & \begin{array}{cccc} \mathrm{NB} & \mathrm{THEN} & \Delta \eta & \begin{array}{cc} \mathrm{is} & \mathrm{NB} \end{array} \end{array} \end{array} \end{array} \end{array}\right.$$

To convert the degrees of membership of the output linguistic variable, the center of gravity defuzzification method was used, because it could calculate the best compromise among multiple output terms [25]. Then, the switching gain was acquired using the following equation

(15) $$\eta =M\int_{0}^{t}\Delta \eta dt$$

where *M* (*M*> 0) denotes the scale factor.

**Figure 4:**
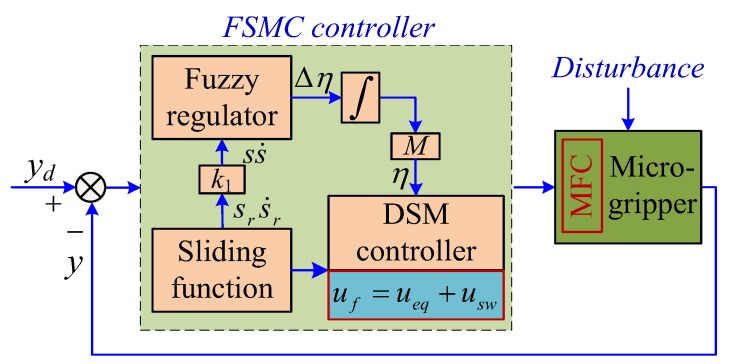
Schematic diagram of the trajectory controller.

Figure 4 shows the schematic diagram of the FSMC for the position control of the MFCmicrogripper.

### 3.2. Force Controller Design for the Left Gripping Arm of the Microgripper

In precise micromanipulation applications, the driving voltages applied to the MFC actuator needed to be regulated once the desired force trajectories of the gripping arm were specified. Considering that the micromanipulation progress is usually very slow [26], and the model errors always exist, a simple proportional-integral (PI) controller was used to compensate the force errors. Based on the force observer, the control algorithm could eliminate the steady error and respond rapidly. The force controller output is given by

(16) $$u_{f}(k)=u_{f}(k-1)+K_{p}[\varepsilon (k)-\varepsilon (k-1)]+\frac{K_{p}T_{0}}{T_{i}}\varepsilon (k)$$

where *k* denotes the sampling number; *ε* is the position tracking error between the desired force trajectory and the actual one; *K_p_* and *T_i_* represent the proportional gain and integral time, respectively; and *T*_0_ is the sampling interval and *T*_0_ is equal to 0.0005 s. 

### 3.3. Hybrid Position/Force Controller Design for the MFC Microgripper

In this section, the hybrid position and force control of the two gripping arms of the MFC microgripper are considered. To carry out a precision micromanipulation application, a procedure similar to the studies in [10,17] was used. To begin with, the two end-effectors were in contact with the micro-object. Then, several concurrent position and force trajectories were used to grip the object. After that, the desired position and force return to zero to release the micro-object.

**Figure 5:**
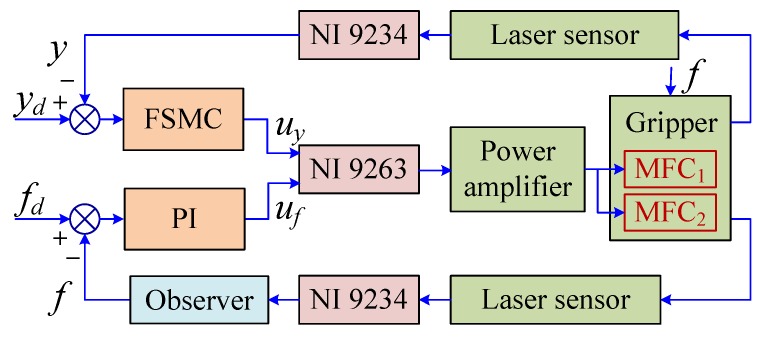
Schematic diagram of the hybrid position/force control scheme.

Finally, the FSMC for the position and the PI controller for the gripping force were used together. The output of the right and the left gripping arms were measured by the laser sensor. Then, the gripping force of the left gripping arm was estimated using a popular force observer (see Section 4.3). Figure 5 gives the schematic diagram of the hybrid position and force control scheme.

## 4. Experimental Results and Discussions

### 4.1. Prototype Development

**Figure 6:**
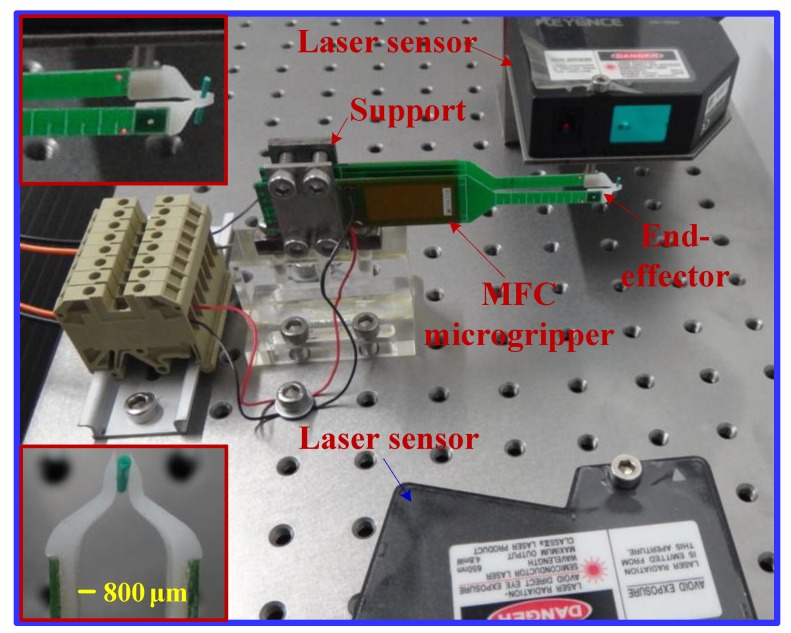
Photograph of the experimental setup.

The prototype and the experimental setup of the MFC microgripper are presented in Figure 6. Two MFC actuators (model: M2814-P1, from Smart Material Corp) were driven by a power amplifier (model: PZD700A, from Trek Corporation, Waterloo, WI, USA). The power amplifier had a maximal voltage of 700 V and an amplification ratio of 70. A laser displacement sensor (model: LK-G30, from Keyence Corporation, Osaka, Japan) was used to measure the output displacement of the MFC microgripper, which had a 50-nm resolution within 10 mm. As the range of the displacement was at the micron level, while the range of the gripping force was at the millinewton level, another laser sensor (model: LK-G150, from Keyence Corporation) with a 500-nm resolution was used to evaluate the force of the MFC microgripper. Moreover, four-channel A/D and D/A modules for data acquisition and control output were provided by a National Instruments (NI) cDAQ-9174 chassis in combination with an analog input module NI-9234 and an analog output module NI-9263. Furthermore, the control system was implemented using a personal computer (PC) with Labview software. In addition, a wire of the single-mode optical fiber (the diameter is 750 μm) was chosen as the micro-object to be manipulated, as illustrated in the magnified view in Figure 6. 

### 4.2. Output Displacement Test

**Figure 7:**
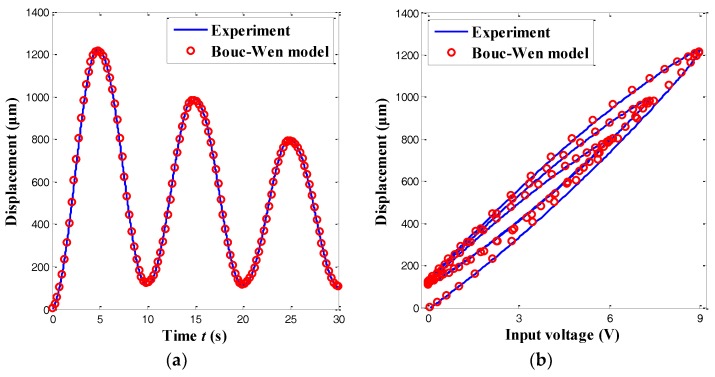
Experimental result of gripping range by applying a multi-amplitude signal: (**a**) time history results; and (**b**) hysteresis loops.

Using alaser displacement sensor, the output displacement of the MFC microgripper can be experimentally measured. Due to the symmetric structure, the output displacement was tested by exerting amulti-amplitude sine-wave voltage (ranging from 0 to 9 V) to the MFC actuator of the rightgripping arm, as illustrated in Figure 7. The maximal output displacement of one gripping arm was approximately 1221.3 μm. When compared with the reported microgrippers in [10,11,17,19], the proposed MFC one presented a much larger output displacement, which is preferable for multiscale micromanipulation tasks. Moreover, significant hysteresis loops, which arose from the inherent characteristics of the piezoelectric actuators, are exhibited in Figure 7b. It was observed that the maximal hysteresis accounted for 17.3% of the output displacement. To compensate for the piezoelectric hysteresis in the assembly and manipulation tasks, a precise control strategy for the MFC microgripper was required.

### 4.3. Force Observer Development

To estimate the force signal in the microgripper, various force observers have been developed. For example, a linear force observer using a simple dynamic model is reported in [27]. However, this linear observer exhibited a low accuracy, owing to the piezoelectric hysteresis and creep phenomenon. After that, a nonlinear force estimator combining the hysteresis and creep models was proposed in [28]. Nevertheless, model errors are inevitable. Hence, a more effective force observer was developed [29] that could be carried out without the hysteresis and creep models. In previous work [29], a fourth-order model was employed to construct the force observer. Alternately, a simple second-order model could also be used in the recent work [17].

In this paper, a force observer in [17] was used to estimate the gripping force between the left gripping arm and the micro-object. The force observer in the time domain is given by

(17) $$F(t)=\frac{1}{s_{p}}L^{-1}\left\{G^{-1}\left(s\right)\right\}\left[y\left(t\right)-y_{1}\left(t\right)\right]-\frac{d_{p}}{s_{p}}\left[u\left(t\right)-u\left(t-T\right)\right]$$

where *s_p_*denotes the elastic constant of the gripping arm; *L*^−1^ is the inverse Laplace transform operator; *G* (*s*) is the plant model (with unity DC gain) under the excitation of the driving voltage; *y* is the position output excited by the driving voltage *u* and the gripping force *F*, whereas *y*_1_represents the position output under the excitation of the driving voltage *u* alone; and *u*(*t*) and *u*(*t*−*T*) are the driving voltage at the time instances *t* and *t*−*T*.

**Figure 8:**
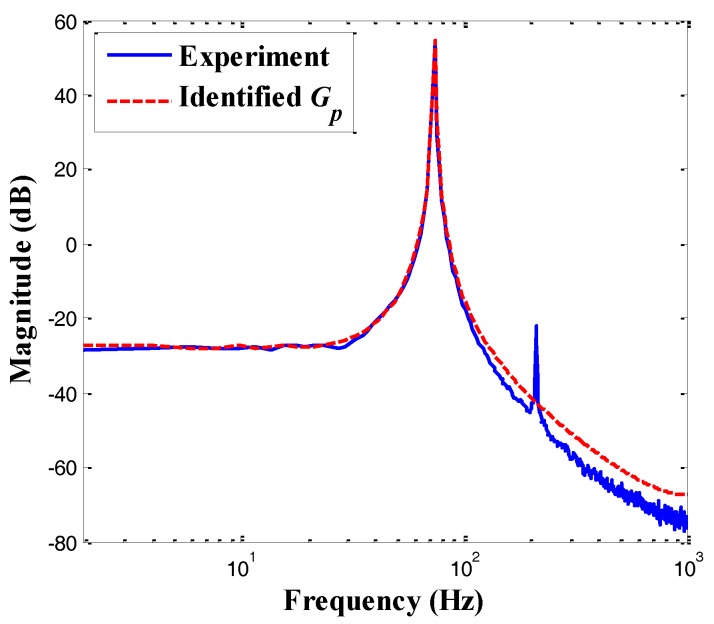
Frequency response of the gripping system.

The plant model can be identified using the sweep-sine method when the excitation voltage is applied to the left gripping arm alone. Figure 8 shows the frequency response of the microgripper derived from the spectral analysis. As shown in Figure 8, the identified second-order model was consistent with the system dynamics in low frequencies. Moreover, the microgripper had a first natural frequency of 74.2 Hz and a second natural frequency of 211.5 Hz. According to recent studies [30,31], the effect of the high-frequency modes was usually very small, and the dynamic characteristics of the flexible structure mainly depended on its first transverse bending mode; thus, the higher modes could be ignored. Thus, only the first mode was selected (i.e., a second-order model) to demonstrate the feasibility of the designed control scheme in this paper. 

The elastic constant *s_p_* was determined by hanging a known weight (10 mN) and acquiring the caused displacement, i.e., *s_p_* = 40.32 μm/mN. Moreover, the piezoelectric coefficient *d_p_*was identified as the DC gain (i.e., *d_p_* = 132.9 μm/V). Then, the plant model *G* with unity dc gain could be derived by *G* = *G_p_*/*d_p_*, which is given by

(18) $$G(s)=\frac{2.174\times 10^{5}}{s^{2}+8.609s+2.174\times 10^{5}}$$

**Figure 9:**
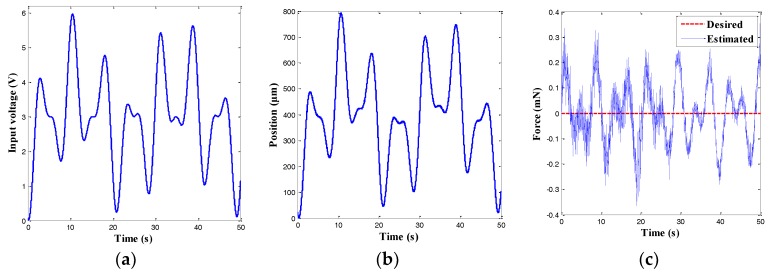
Estimation results of the force observer without gripping force. (**a**) Input voltage; (**b**) Output displacement; and (**c**) Force signal.

In addition, to evaluate the characteristics of the force observer, the input voltage shown in Figure 9a was exerted to the power amplifier to drive the left MFC actuator, while the external force was set to be zero. The measured position and force are depicted in Figure 9b,c, respectively. It was found that the maximal error of the force observer was approximately 0.354 mN, which indicated that the gripping force could be accurately estimated. 

### 4.4. Experimental Results for the Hybrid Position/Force Control

#### 4.4.1. Experimental Results for the Single Position Control

**Figure 10:**
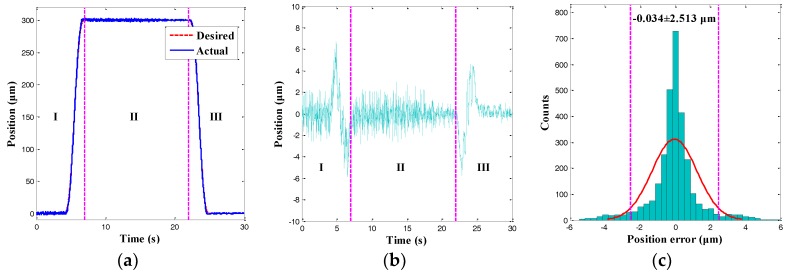
Position control results of a cycloid trajectory. (**a**) Position tracking result; (**b**) Position tracking error; and (**c**) Histogram of position tracking error.

In this section, only the single position control of the right gripping arm for the microgripper was performed. For various practical micromanipulation operations, the overall manipulation process can be classified into three phases: the gripping phase (I), the holding phase (II), and the releasing phase (III). Accordingly, the trapezoidal trajectory is usually used in such applications. However, this trajectory also presents an unwanted overshot at the turning points, which would damage the fragile micro-objects [13]. Hence, an improved cycloid trajectory was proposed to suppress the overshoot, as shown in phase I and phase III in Figure 10. As illustrated in Figure 10, the desired trajectory was accurately tracked in the three phases. In the gripping phase (I), the gripping arm moved as the driving voltages increased. In the holding phase (II), the position trajectory was kept at 300 μm. In the releasing phase (III), the trajectory returned to zero. As the position error was random, the normally distributed tracking result demonstrated the reliability of the position tracking result. Moreover, the cycloid tracking error presented an approximately normal distribution, and the 95% confidence interval was−0.034 ± 2.513 μm. Therefore, the RMSE of the cycloid trajectory tracking error was calculated as 1.282 μm. The value was 0.43% smaller than the maximal amplitude of the desired trajectory. The experiments indicated that the output displacement of the right gripping arm could be precisely tracked using the position control scheme.

#### 4.4.2. Experimental Results for the SingleForce Control

**Figure 11:**
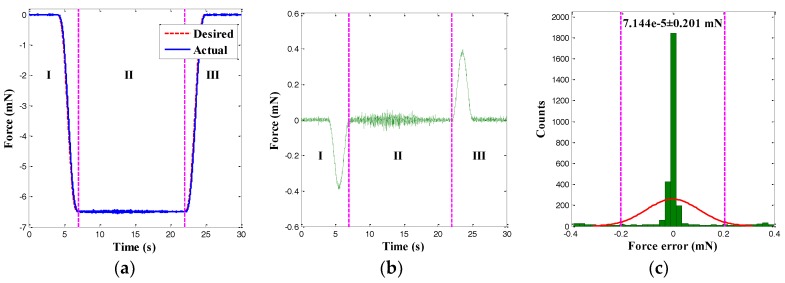
Force control results of a cycloid trajectory. (**a**) Force tracking result; (**b**) Force tracking error; and (**c**) Histogram of force tracking error.

In this section, only the single force control of the left gripping arm for the microgripper was performed. According to the force control strategy, the input voltages of the left MFC actuator were regulated. To begin with, acycloid force trajectory with a 6.5-mN amplitude was applied to the left gripping arm. The corresponding tracking result of the gripping force is presented in Figure 11. It was observed that the actual force tracked the desired force trajectory accurately. Moreover, the trajectory tracking error and its histogram are depicted in Figure 11b,c. It was found that the force tracking error presented a normal distribution and that the 95% confidence interval was 7.144×10^−5^ ± 0.201 mN. Since the force error was random, the normally distributed tracking result demonstrated the reliability of the force tracking result. Furthermore, the arbitrary force tracking error had a RMSE of 0.103 mN, which was 1.58% smaller than the maximum of the arbitrary force trajectory. Thus, the MFC microgripper also presented good force control accuracy. 

#### 4.4.3. Experimental Results for the Hybrid Position/Force Control

**Figure 12:**
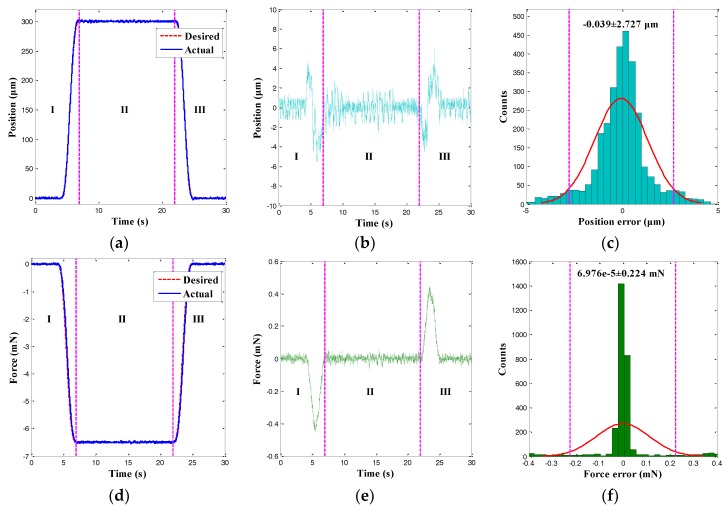
Hybrid position/force control results of a cycloid trajectory. (**a**) Position tracking result; (**b**) Position tracking error; (**c**) Histogram of position tracking error; (**d**) Force tracking result; (**e**) Force tracking error; and (**f**) Histogram of force tracking error.

In this section, the hybrid position/force control of the microgripper was investigated. In particular, the FSMC (see Figure 4) for the position and the PI controller for the force of the microgripper were simultaneously used. First, the cycloid trajectory was tracked, and the control results are shown in Figure 12. It was observed that the actual output displacement and gripping force could track the desired trajectories accurately and the significant hysteresis as the properties of the MFC actuator was reduced to a negligible level. Moreover, the histograms for the position and the force tracking errors are presented in Figure 12c,f. The concurrent arbitrary trajectories tracking errors exhibited normal distributions, and the 95% confidence intervals of the position and the force tracking errors were −0.039 ± 2.727 μm and 6.976×10^−5^ ± 0.224 mN. Hence, the RMSEs of the position and force tracking errors were calculated as 1.391 μm and 0.114 mN, which were 0.46% and 1.76% smaller than the peak-to-peak amplitude of the desired trajectories. The MFC microgripper presented good position and force control accuracy simultaneously.

**Figure 13:**
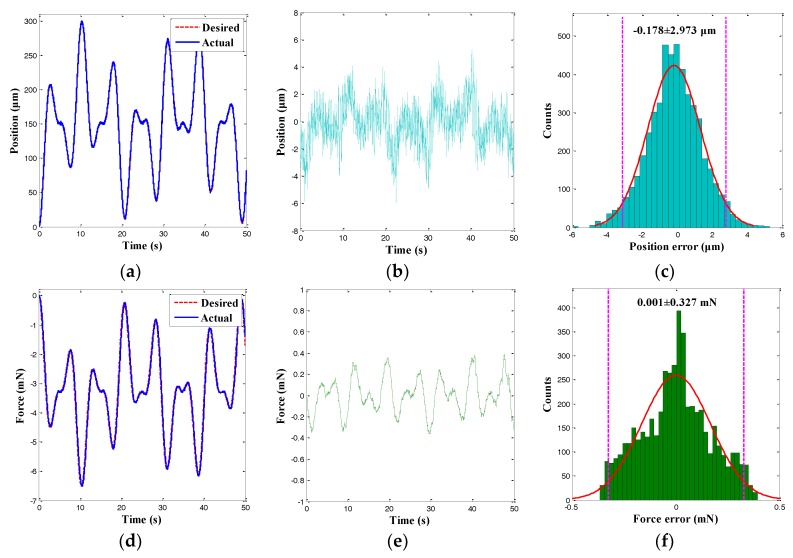
Hybrid position/force control results of an arbitrary trajectory. (**a**) Position tracking result; (**b**) Position tracking error; (**c**) Histogram of position tracking error; (**d**) Force tracking result; (**e**) Force tracking error; and (**f**) Histogram of force tracking error.

To further demonstrate the control performance of the FSMC, another tracking experiment with an arbitrary trajectory was performed, and the corresponding control results are depicted in Figure 13. It was observed that the position and force tracking errors of the trajectory presented approximately normal distributions. Furthermore, the 95% confidence interval of the position tracking errors was −0.178 ± 2.973 μm, and the 95% confidence interval of the force tracking errors was 0.001 ± 0.327 mN. Hence, the RMSEs of the cycloid trajectory were calculated to be 1.517 μm and 0.167 mN, respectively. The values were 0.51% and 2.57% smaller than the peak-to-peak amplitude of the desired trajectories. Experiments demonstrated that the MFC microgripper exhibited good position and force control accuracy simultaneously. Therefore, the hybrid position and force control scheme adopted for the MFC microgripper was feasible and effective. 

### 4.5. Discussion

**Figure 14:**
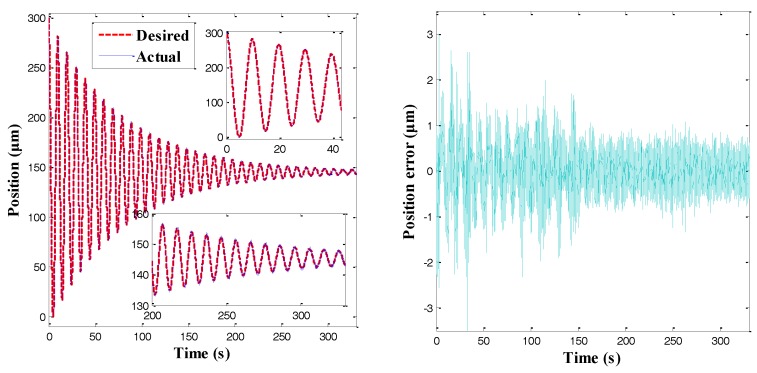

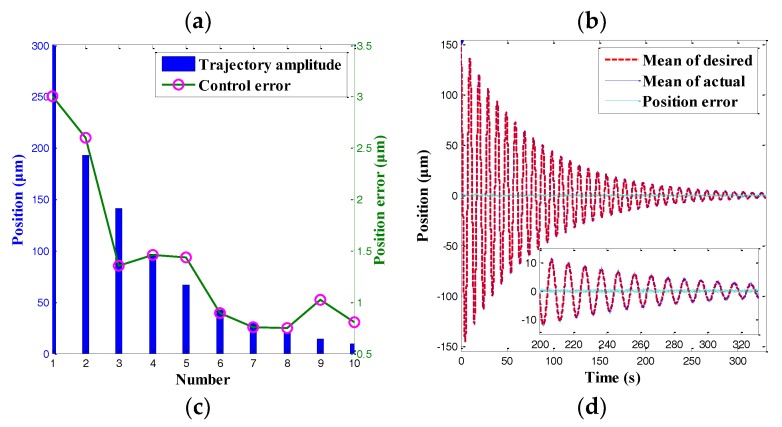
Position control results of a multi-amplitude trajectory. (**a**) Position tracking result; (**b**) Position tracking error; (**c**) Position error versus trajectory amplitude; and (**d**) Position error versus mean of the desired trajectory.

While the displacement obtained by one of the arms was indeed higher than the other piezoelectric drives, it was still necessary to demonstrate that the gripper could be used to manipulate small-scale objects (about 10 microns or smaller in size). Thus, a sinusoidal position trajectory with multiple amplitudes was applied to the microgripper. The corresponding tracking results are shown in Figure 14. It was observed that the actual position tracked the desired force trajectory accurately, as illustrated in Figure 14a. Moreover, Figure 14b,c shows that the control error basically decreased with the trajectory amplitude, and the maximal control error was approximately 0.6 μm when the trajectory amplitude was 10 μm. In fact, the control accuracy was heavily affected by the detection resolution of the microgripper system [12]. Thus, the gripper could be used to manipulate small scale-objects. Furthermore, the position tracking error was plotted together with the mean of the desired trajectory in Figure 14a. According to Figure 14d, the plots of the signal/noise for a wide range of displacements are presented. Accordingly, the developed microgripper could be used for multiscale manipulation.

**Figure 15:**
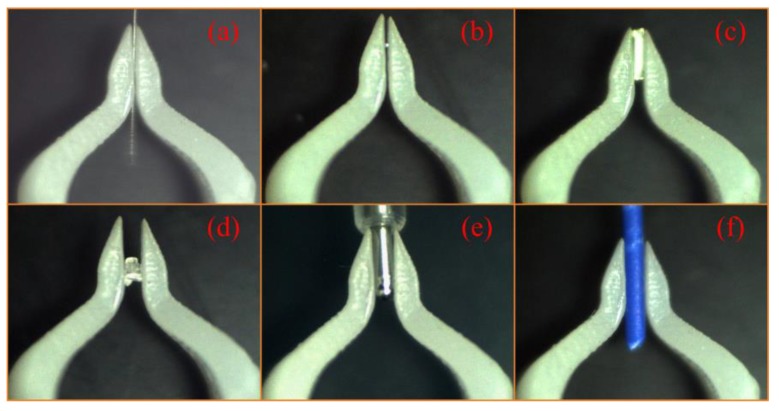
Cases of micromanipulation tasks: (**a**) holding a single mode fiber of 125 μm; (**b**) manipulation of a 200 μm diameter solder ball; (**c**) manipulation of a 440 μm resistance; (**d**) clamping an irregular crystal of 550 μm; (**e**) manipulation of a 700 μm microcomponent; and (**f**) manipulating a wire cable of 860 μm.

In order to demonstrate that the microgripper could handle multiscale micro-objects, several typical dimensions of micro-objects between 100 μm to 900 μm were used to perform manipulation tasks, as shown in Figure 15.

Table 1 summarizes the performances of the developed MFC microgripper. According to Table 1, the MFC microgripper exhibited a large workspace range and high position/force control accuracy. Hence, the microgripper with the designed hybrid control scheme was suitable for use inprecision micromanipulation. Furthermore, the input voltage employed in this study was 50% less than its nominal maximum voltage (−500 V~1500 V) and only one MFC actuator was glued onto each gripping arm, so the MFC microgripper was capable of multiscale micromanipulation and microassembly tasks. Nevertheless, a higher resonant frequency was expected to derive a much larger operation bandwidth [32].

In addition, the MFC microgripper also had a large global size, as shown in Table 2. It is well known that the output displacement of the microgripper has to be evaluated with its total structural dimensions. Therefore, for a clear view, the displacement-volume ratios of the reported piezoelectric microgrippers [10,11,17,19] were compared in Table 2. It was observed that the developed MFC microgripper outperformed the others in terms of having a higher displacement-volume ratio and a larger gripping range. Moreover, the manipulation accuracy of the microgripper could be guaranteed by the hybrid control scheme. 

## 5. Conclusions

The above sections presented the development, implementation, and hybrid position/force control of a new MFC microgripper with a large displacement-volume ratio. Based on the MFC actuator, the proposed microgripper was actuated and designed. Through the FSMC, precision position control of the microgripper was obtained. Meanwhile, gripping force control was guaranteed by the PI controller. Note that the microgripper was driven by two independent MFC actuators; the right actuator was used to position the micro-object and the left one was used to control the force. A series of experimental investigations were carried out to test the characteristics of the microgripper and to validate the designed hybrid control scheme. The main conclusions of this study can be summarized as follows:

(1) The proposed MFC microgripper presented a large output displacement and a high displacement-volume ratio, which demonstrated that the microgripper was capable of multiscale micromanipulation;
(2) The designed hybrid control scheme, which employed the FSMC combined with the PI controller, was feasible. The control scheme was able to regulate both the position and the gripping force simultaneously, and its effectiveness and simplicity make it suitable for industry systems.

In the future, other force sensing techniques will be performed to achieve a more compact size. Moreover, parameter optimization will be conducted to obtain a higher natural frequency.

## Figures and Tables

**Table 1:** Performances of the MFC microgripper.

Parameter	Value
Dimension	86.8 mm×10.8 mm×(20−5)mm
Output displacement	1221.3 μm
First resonant frequency	74.2 Hz
Arbitrary position/force RMSEs	1.517 μm/0.167 mN
Relative RMSEs (Arbitrary)	0.51%/2.57%
Cycloid position/force RMSEs	1.391 μm/0.114 mN
Relative RMSEs (Cycloid)	0.46%/1.76%

**Table 2:** Comparisons with reported piezoelectric microgrippers.

No.	Actuation Principle	Output Displacement	Displacement-Volume Ratio	Control Variables	Independent Regulation	Relevant Literature
1	Piezoelectric bimorph	20 μm	0.049 μm∙mm^−3^	Both	No	[17]
2	Thermo-piezoelectric	80 μm	0.003 μm∙mm^−3^	Both	Yes	[10]
3	Piezoelectric stack	328.2 μm	0.016 μm∙mm^−3^	Both	Yes	[19]
4	Piezoelectric stack	427.8μm	0.019 μm∙mm^−3^	---	---	[11]
5	MFC actuator	1212.4 μm	0.101 μm∙mm^−3^	Both	Yes	Current

## References

[1] Bharanidaran R., Ramesh T. (2017). A modified post-processing technique to design a compliant based microgripper with a plunger using topological optimization. Int. J. Adv. Manuf. Technol..

[2] Li P., Gu H.C., Song G.B., Zhang R., Mo Y.L. (2010). Concrete structural health monitoring using piezoceramic-based wireless sensor networks. Smart Struct. Syst..

[3] El-Sayed A.M., Abo-Ismail A., El-Melegy M.T., Hamzaid N.A., Abu Osman N.A. (2013). Development of a micro-gripper using piezoelectric bimorphs. Sensors.

[4] Sethi V., Franchek M.A., Song G.B. (2011). Active multimodal vibration suppression of a flexible structure with piezoceramic sensor and actuator by using loop shaping. J. Vib. Control.

[5] Sethi V., Song G.B. (2008). Multimodal vibration control of a flexible structure using piezoceramic sensor and actuator. J. Intell. Mater. Syst. Struct..

[6] Zhang T., Li H.G., Zhong Z.Y., Cai G.P. (2015). Hysteresis model and adaptive vibration suppression for a smart beam with time delay. J. Sound Vib..

[7] Pandey A., Arockiarajan A. (2017). An experimental and theoretical fatigue study on macro fiber composite (MFC) under thermo-mechanical loadings. Eur. J. Mech. A-Solids.

[8] Wang D.H., Yang Q., Dong H.M. (2013). A monolithic compliant piezoelectric-driven microgripper: Design, modeling, and testing. IEEE-ASME Trans. Mech..

[9] Ai W.J., Xu Q.S. (2014). Overview of flexure-based compliant microgrippers. Adv. Robot. Res..

[10] Rakotondrabe M., Ivan I.A. (2011). Development and force/position control of a new hybrid thermo-piezoelectric microgripper dedicated to micromanipulation tasks. IEEE Trans. Autom. Sci. Eng..

[11] Yang Y.L., Wei Y.D., Lou J.Q., Tian G., Zhao X.W., Fu L. (2015). A new piezo-driven microgripper based on the double-rocker mechanism. Smart Mater. Struct..

[12] Sun X.T., Chen W.H., Tian Y.L., Fatikow S., Zhou R., Zhang J.B., Mikczinski M. (2013). A novel flexure-based microgripper with double amplification mechanisms for micro/nano manipulation. Rev. Sci. Instrum..

[13] Yang Y.L., Wei Y.D., Lou J.Q., Fu L., Tian G., Wu M. (2016). Hysteresis modeling and precision trajectory control for a new MFC micromanipulator. Sens. Actuator A-Phys..

[14] Komati B., Clevy C., Lutz P. (2016). High bandwidth microgripper with integrated force sensors and position estimation for the grasp of multistiffnessmicrocomponents. IEEE-ASME Trans. Mech..

[15] Xu Q.S. (2014). Design and smooth position/force switching control of a miniature gripper for automated microhandling. IEEE Trans. Ind. Electron..

[16] Wang F.J., Liang C.M., Tian Y.L., Zhao X.Y., Zhang D.W. (2016). Design and control of a compliant microgripper with a large amplification ratio for high-speed micro manipulation. IEEE-ASME Trans. Mech..

[17] Xu Q.S. (2013). Precision position/force interaction control of a piezoelectric multimorph microgripper for microassembly. IEEE Trans. Autom. Sci. Eng..

[18] Xu Q.S. (2015). Robust impedance control of a compliant microgripper for high-speed position/force regulation. IEEE Ind. Electron..

[19] Yang Y.L., Wei Y.D., Lou J.Q., Xie F.R., Fu L. (2016). Development and precision position/force control of a new flexure-based microgripper. J. Micromech. Microeng..

[20] Qiu Z.C., Zhang S.M. (2017). RBF neural network-based sliding mode vibration control of a flexible cantilever plate using laser displacement measurement. Proc. Inst. Mech. Eng. Part G-J. Aerosp. Eng..

[21] Song G.B., Gu H. (2007). Active vibration suppression of a smart flexible beam using a sliding mode based controller. J. Vib. Control.

[22] Qiu Z.C., Zhang S.M. (2016). Fuzzy fast terminal sliding mode vibration control of a two-connected flexible plate using laser sensors. J. Sound Vib..

[23] Hassani V., Tjahjowidodo T., Do T.N. (2014). A survey on hysteresis modeling, identification and control. Mech. Syst. Signal Proc..

[24] Li L.Y., Song G.B., Ou J.P. (2010). Nonlinear structural vibration suppression using dynamic neural network observer and adaptive fuzzy sliding mode control. J. Vib. Control.

[25] Qiu Z.C., Wang B., Zhang X.M., Han J.D. (2013). Direct adaptive fuzzy control of a translating piezoelectric flexible manipulator driven by a pneumatic rodless cylinder. Mech. Syst. Signal Proc..

[26] Zhang D.P., Zhang Z.T., Gao Q., Xu D., Liu S. (2014). Development of a monolithic compliant SPCA-driven micro-gripper. Mechatronics.

[27] Haddab Y., Chaillet N., Bourjault A. A microgripper using smart piezoelectric actuators. Proceedings of the 2000 IEEE/RSJ International Conference on Intelligent Robots and Systems.

[28] Rakotondrabe M., Haddab Y., Lutz P. Nonlinear modeling and estimation of force in a piezoelectric cantilever. Proceedings of the 2007 IEEE/ASME International Conference on Advanced Intelligent Mechatronics.

[29] Xu Q.S. A new method of force estimation in piezoelectric cantilever-based microgripper. Proceedings of the 2012 IEEE/ASME International Conference on Advanced Intelligent Mechatronics (AIM).

[30] Li L.Y., Song G.B., Ou J.P. (2013). A nonlinear structural experiment platform with adjustable plastic hinges: Analysis and vibration control. Smart Struct. Syst..

[31] Yang Y.L., Wei Y.D., Lou J.Q., Fu L., Zhao X.W. (2017). Nonlinear dynamic analysis and optimal trajectory planning of a high-speed macro-micro manipulator. J. Sound Vib..

[32] Gu G.Y., Zhu L.M., Su C.Y., Ding H., Fatikow S. (2016). Modeling and control of piezo-actuated nano-positioning stages: A survey. IEEE Trans. Autom. Sci. Eng..

